# Modulation of gamma and alpha spinal motor neurons activity by trans‐spinal direct current stimulation: effects on reflexive actions and locomotor activity

**DOI:** 10.14814/phy2.12696

**Published:** 2016-02-11

**Authors:** Zaghloul Ahmed

**Affiliations:** ^1^ Department of Physical TherapyCollege of Staten Island for Developmental NeuroscienceThe College of Staten IslandStaten IslandNew York; ^2^ Graduate Center/The City University of New YorkNew YorkNew York

**Keywords:** Alpha motor neuron, gamma, locomotion, trans‐spinal DCS

## Abstract

Spontaneous and evoked spinal activities interact to set the characteristics of emergent motor responses. Gamma motor neurons have feedforward and feedback functions in motor control, which are crucial for transforming motor commands into action. Meanwhile, the intrinsic excitability and functional connectivity of alpha motor neurons determine the accuracy of actions. In this study, we investigated the effects of trans‐spinal direct current stimulation (tsDCS) on spontaneous and cortically evoked activity of well‐isolated single units of gamma and alpha motor neurons in mice. We also investigated the effects of tsDCS on reflexive and locomotor actions. In general, motor neurons showed increased responses to cathodal tsDCS (c‐tsDCS) and decreased responses to anodal tsDCS (a‐tsDCS). These effects were observed for cortically evoked discharges and spontaneous firing rates of gamma motor neurons, cortically evoked discharges of larger alpha motor neurons, and spontaneous firing rates of smaller alpha motor neurons. An exception was that spontaneous firing rates of larger alpha motor neurons showed the opposite pattern of reduction by c‐tsDCS and increase by a‐tsDCS. Reflexive and voluntary behavior were also increased by c‐tsDCS and reduced by a‐tsDCS. Specifically, the amplitude and duration of crossed and tail pinch reflexes in decerebrate animals and the quality of ground and treadmill walking patterns in healthy awake animals showed this pattern. These polarity‐specific changes in behavior could be attributed to polarity‐mediated modulation of alpha and gamma motor neuron activity and spinal circuitry. The results reveal an important principle: effects of tsDCS on spinal motor neurons depend on current polarity and cell size.

## Introduction

Direct current stimulation (DCS) offers a new method to manipulate ongoing behavior, subsequent plasticity, and learning. For example, anodal trans‐spinal DCS (a‐tsDCS) increases the spontaneous firing rate of motor neurons and reduces their evoked responses. Cathodal trans‐spinal DCS (c‐tsDCS) does the opposite. This DC action changes the magnitude and direction of spinal plasticity (Aguilar et al. 2011; Ahmed 2011). Spinal DCS modulates synaptic background and evoked activity in a similar way. That is, hyperpolarizing DC reduces the frequency of spontaneous postsynaptic potentials (Hubbard and Willis 1962a) and increases evoked postsynaptic potentials (Del Castillo and Katz 1954; Eccles et al. 1962). Depolarizing current does the opposite (Hubbard and Willis 1962b, 1968). Thus, we deduce that DCS manipulates the spontaneous‐to‐evoked activity ratio. According to new learning theories, this ratio determines the learning rules (Toyoizumi et al. 2013). Specifically, increasing the spontaneous‐to‐evoked activity ratio impairs externally driven learning experiences, and decreasing this ratio increases the sensitivity of neurons to externally driven learning experiences. This pattern implies that the modification in this ratio by stimulation could affect the fate and magnitude of motor learning. Spontaneous activity is known to have strong modulatory effects on neural plasticity (Kirkwood et al. 1996). For example, the level of spontaneous activity modifies the likelihood of expression of synaptic plasticity, as well as the direction of plasticity (depression or facilitation) (Crochet et al. 2006). Spontaneous activity also affects neuronal membrane excitability (Rosenkranz 2011). Thus, spontaneous activity could change responsiveness and functional connectivity of neurons, which in turn would affect behavior. In the spinal cord, background activity plays a role in synaptic plasticity (Fedirchuk et al. 1999; Gonzalez‐Islas and Wenner 2006). In addition, changes in spinal spontaneous activity are an important factor in pathological conditions, such as spasticity (Mottram et al. 2009) and pain (Bedi et al. 2010).

Gamma motor neurons apply feedforward control (Sjostrom and Zangger 1975; Dimitriou and Edin 2010), as well as online feedback control (Vallbo 1981), to sensory information to predict and adjust motor commands. These functions imply that gamma motor neuron activity plays an important role in motor control and learning. Muscle spindles relay sensory information about the relative positions of body segments to the central nervous system. Gamma motor neurons adjust the sensitivity of muscle spindles to allow the associated sensory nerves to signal changes in muscle length at the full range of a joint (Vallbo 1981). Therefore, manipulation of this important system by tsDCS could provide an advantage to motor control and learning approaches. The intrinsic excitability and connectivity of alpha motor neurons affect the final motor output (Kiehn and Eken 1998; Tresch and Kiehn 2002). tsDCS has online (during) and offline (after) modulatory effects on the firing rate of spinal spontaneous activity. However, the nature and functional significance of that activity are not known.

Spinal motor neurons are topographically organized in the rostrocaudal axis in pools and mini columns (see review by Jessell et al. (2011)). Their anatomical organization correlates strongly with their function (Henneman and Mendell 2011). Within the dorso‐ventral axis, spinal neurons are also arranged in laminae, which contain neurons that are anatomically and functionally distinct. Modulation of neurons by tsDCS depends on many factors, such as orientation relative to the direction of current, distance from the electrode, polarity, and direction of the action potential propagation (Ahmed 2014a,b). Thus, anatomically and functionally distinct spinal neurons could be differently modulated by tsDCS. In addition, tsDCS modulates synaptic transmission (Eccles et al. 1962; Hubbard and Willis 1962a,b). Hence, its effects could depend on the type and orientation of presynaptic inputs relative to the direction of current. tsDCS modulates spinal circuit activity, which seems to be state‐dependent (Ahmed 2013a,b). Alpha motor neurons are subdivided based on their size and function. Most of the factors that influence tsDCS modulatory effects can be found in distinct subclasses of spinal motor neurons. Thus, in this study, we hypothesized that tsDCS would differentially affect spontaneous and evoked activity of gamma and alpha motor neurons based on tsDCS polarity and cell type. Moreover, since alpha and gamma motor neurons are the output neurons of the spinal cord, their activity represents the final outcome of spinal network processes, and tsDCS effects would be expected to influence reflexive and locomotor behavior.

We used extracellular recording to test the effects of tsDCS on single units from sciatic nerve branches. Recorded spikes were separated into gamma and alpha motoneurons. The effects of tsDCS on reflexive and locomotor behaviors were tested in decerebrate and healthy animals. These experiments revealed important mechanisms mediating tsDCS effects on spinal and neuromuscular output.

## Materials and Methods

### Animals

Adult male CD‐1 mice (*n *=* *35; weight, 40–55 g) were used for this study. Protocols were approved by the Institutional Animal Care and Use Committee of the College of Staten Island.

### Setup for extracellular single‐unit recording in anesthetized animals

**Figure 1:**
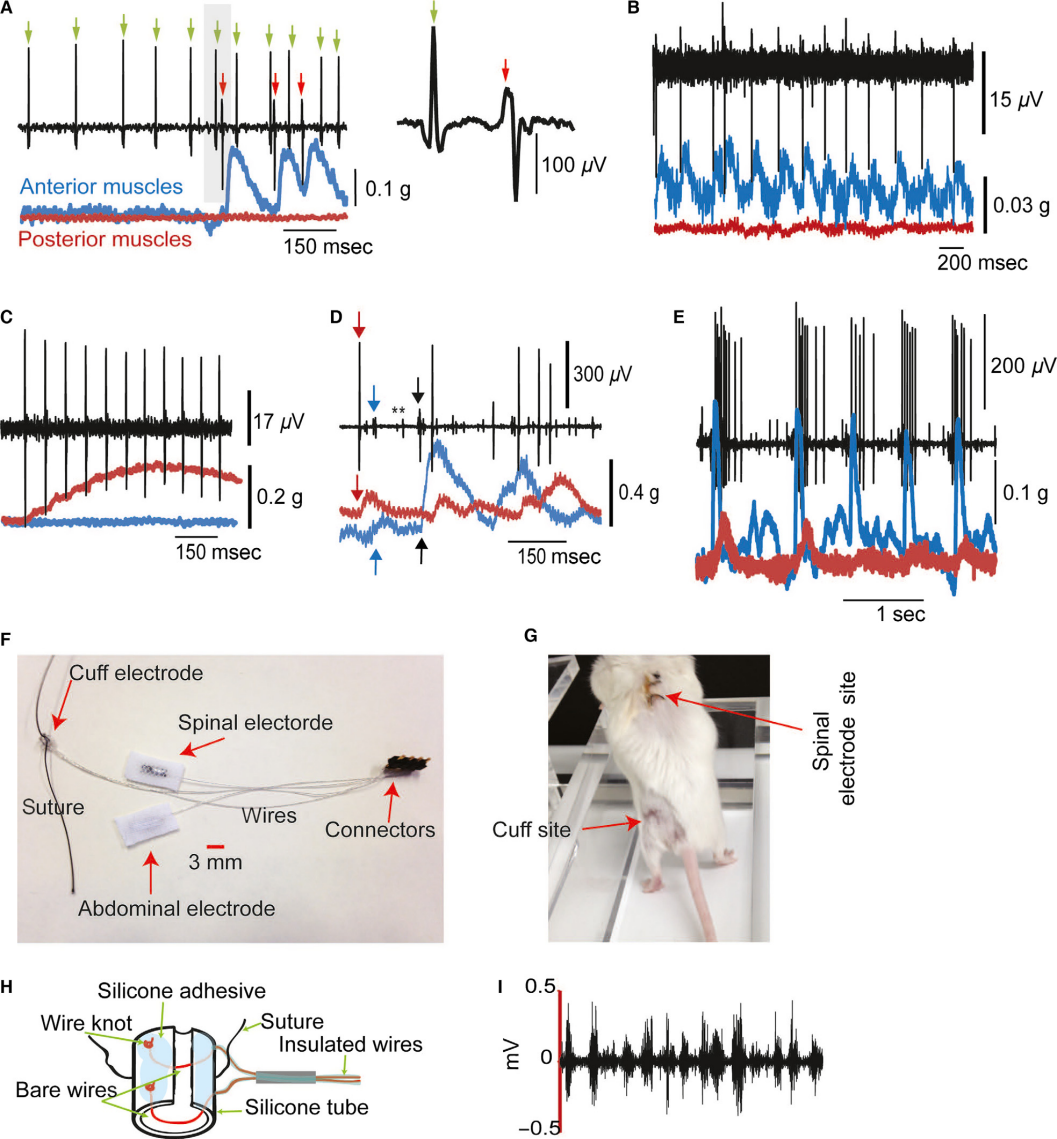
Characterization of spontaneous activity. (A) Nerve and muscle traces showing examples of a gamma motor neuron (green arrow) and alpha motor neuron (red arrow). Inset on the right is an enlargement of the shaded area. (B) Example of a motor neuron from the anterior compartment muscles. (C) Example of a tonic motor neuron from the posterior compartment muscles. (D) Example of raw traces to show overlapping of five motor neurons (two gamma [asterisks] and three alpha [arrows]) recorded with the same electrode. (E) Example of rhythmic gamma and alpha motor neurons. Note that the larger sizes belong to gamma neurons, and the smaller spikes are from alpha neurons. (F) Recording and stimulating implantable system in awake animals. (G) Animal standing and walking on a treadmill with no abnormalities. Note that the purpose of this image is to show that the animal can move freely with the implant. During testing, mice were walking in quadrupedal manner. (H) Cuff electrode. (I) Raw traces of nerve activity recorded by the nerve cuff during treadmill walking.

Animals were anesthetized using ketamine/xylazine (90/10 mg/kg, i.p.), and anesthesia was kept at a moderate level throughout experiments. Each animal was placed in a mouse stereotaxic apparatus, which was placed in a custom‐made spinal‐column clamping system. The bone at the base of the tail was fixed to the base of the system with surgical pins. Holes were made in the distal parts of the femur and tibia, and nails were inserted to fix these bones to the base. Incisions were made in the skin covering the vertebral column (from midthoracic to sacral region) and left hindlimb, and the skin was moved to the side and held with clips. The posterior muscles (triceps surae, plantaris, flexor hallucis longus, flexor digitorum longus, and tibialis posterior) were carefully separated from the surrounding tissue. The tendons of this group were connected to a force transducer that was connected to a bridge amplifier (Kent Scientific Corporation, Torrington, CT). Similarly, the anterior leg compartment muscles (tibialis anterior, extensor digitorum longus, extensor halluces longus, and fibularis tertius) were isolated and connected to another force transducer. Tension was applied on muscle groups so they respond optimally. Optimal muscles responses were also tested by stimulating the sciatic nerve branches before starting the experiment. Baseline muscle tension was continuously monitored. The sensitivity of the force transducers was adjusted to record single twitch responses (see Fig. 1). Tissue surrounding the distal part of the sciatic nerve was removed, and the sciatic nerve was separated into the tibial and peroneal nerves branches. A craniotomy was made over the contralateral primary motor cortex (M1; 1 mm posterior to bregma and 1 mm lateral to midline) without breaching the dura. The cortex was stimulated with a monopolar electrode (150‐*μ*m tip), and an active electrode was situated on M1. An alligator clip attached to a flap of scalp skin on the frontal aspect of the skull served as the reference electrode. Extracellular recording electrodes (pure iridium, 1–2‐*μ*m tips; 5‐MΩ impedance) were inserted into the sciatic nerve branches. The sciatic nerve and muscle groups were covered with a mixture of silicone oil and petroleum jelly (Vaseline). All extracellular recordings were passed through a high impedance headstage, amplified (Neuro Amp EX, ADInstruments), filtered (100‐Hz–5‐kHz bandpass filter), digitized at 2 kHz, and stored in the computer for further processing. A PowerLab data acquisition system and LabChart 7 software (ADInstruments, Colorado Springs, CO) were used to acquire and analyze the data.

#### Direct current stimulation setup

A Grass stimulator with current isolation unit (DC mode) was used as the current source. The spinal DCS electrode consisted of a small stainless‐steel plate (1.5‐mm width; 3‐mm length; 50 *μ*m thickness) that was sandwiched between silicone rubber (178‐*μ*m thickness) and soft cotton‐wick fabric (0.5 mm thickness). The three layers were bonded together using silicone adhesive and left overnight to dry before use. The final DCS electrode (10 mm wide and 15 mm long) was placed over the T13‐L6 vertebrae. Current strength used in all experiments was 0.5 mA, producing a current density of 3.3 A/m^2^. The reference electrode was an alligator clip that was connected to the abdominal skin. This electrode configuration was previously demonstrated to deliver localized current (Ahmed 2013b; Song et al. 2015).

**Figure 2:**
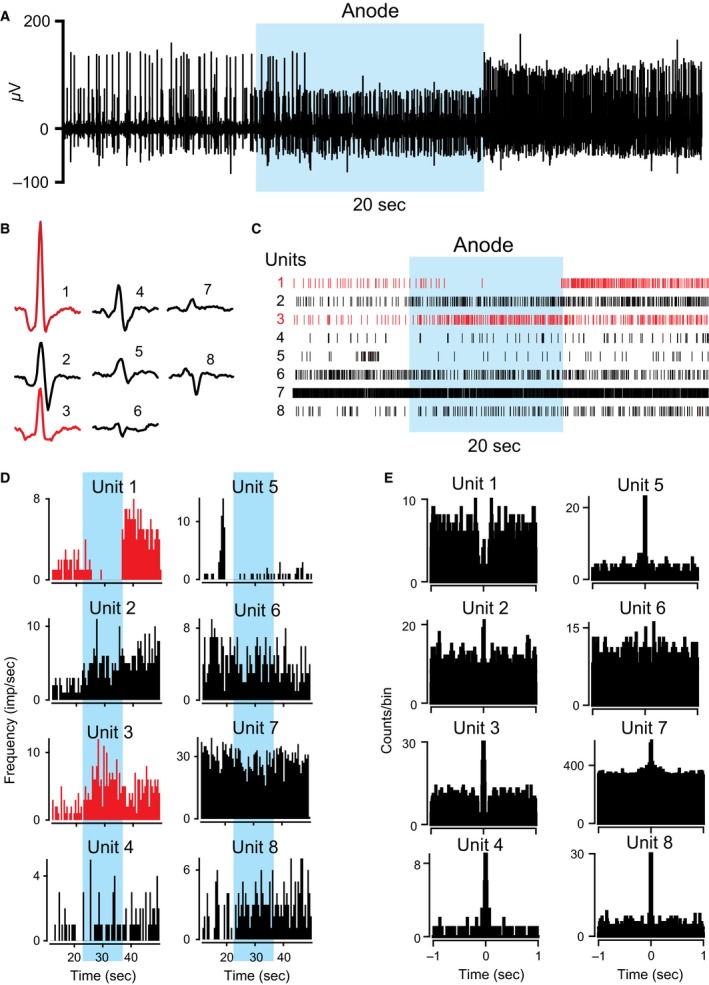
Effects of a‐tsDCS on gamma and alpha motor neuron ensemble. This ensemble of eight active neurons, marked by numbers 1–8, was recorded by one electrode. (A) Raw trace of neuronal activity. (B) Neuronal spikes. (C) Spike rasters showing discriminated spikes. The unit numbers in (C) correspond to the numbers in (B). Red and black colored spikes and rasters were produced by gamma and alpha motor neurons, respectively. (D) Firing rate histograms. Note that firing activity of unit 1 was completely abolished by a‐tsDCS. (E) Autocorrelograms. Units 1 and 6 had regular activity patterns, whereas all other cells had bursting activity patterns. Note that unit 8 had a rhythmic pattern.

Units were identified as gamma or alpha motor neurons (Fig. 1A–E). The most accurate criteria used to identify spikes from alpha motor neurons were their direct relationships with muscle twitches. In general, we used the following eight criteria to identify gamma motor neuron spikes: (1) They fire continuously at a steady rate when muscles are at rest (no stretch or contraction), sometimes for 30 mins (Lund et al. (1979); (2) They have relatively high frequency firing (Lund et al. 1979; Murphy et al. 1984; Murphy 2002); (3) Firing can be evoked by cortical stimulation (see Figs. 5, 6, and 7), as shown by Taylor et al. (2000) in the brain stem; (4) Spikes precede alpha motor neuron activity, but not muscle twitch (Fig. 1A and E); (5) There is no response to passive stretching of the muscle (not shown); (6) Activity is not silenced during the rising phase of muscle contraction (Appenteng et al. 1982; Bessou et al. 1989) (see examples in Fig. 1 A, D, and E, as well as Figs. 5 and 6). In fact, we observed increased activity during the rising phase of twitch force. This argues against an afferent identity of these neurons because afferents would be depressed during the rising phase of the twitch force, according to Appenteng et al. (1982); (7) The strong cross‐correlation between neurons classified as gamma and alpha motor neurons (e.g., Fig. 2) strongly suggests a co‐activation, which argues against an afferent identity of those neurons classified as gamma motor neurons; (8) The shape of the waveform was always different between gamma and alpha motor neurons (see Fig. 1A), which facilitated identification of the neurons for a longer recording period within the same animal. These criteria are shown in examples and were used throughout the identification process. Spike size was not used to characterize neurons because it can vary as a function of distance from the recording electrode. To determine the sizes of alpha motor neurons, we used the sizes and slopes of twitches (compare twitch sizes in Fig. 1A or C to those in Fig. 1D) (Henneman and Olson 1965; McPhedran et al. 1965a,b; Burke 1968; Zajac and Faden 1985; Harris et al. 2006). Peak Analysis software (AdInstrument) was used to calculate slopes and amplitudes of muscle twitches. Spikes were identified based on their characteristics (height, width, and shape) using clustering analysis.

### Decerebrate animals and reflex testing

Animals were anesthetized in a small animal anesthesia system (Kent Scientific) with isoflurane at an initial dose of 5%, followed by 2.5% to maintain deep anesthesia during the procedure. Once anesthetized, animals were placed supine. The trachea was exposed, an incision was made, and an intubation tube was inserted into the trachea opening and secured in place with a suture. The right carotid artery was exposed and ligated. Next, the animal was placed on its ventral aspect. The scalp was shaved, and an incision was made to expose the skull. A large craniotomy was made over the two hemispheres. A precollicular decerebration was accomplished using a blade to make a 45° cut from the posterior aspect of the cortex through the brain to the base of the cranium (Nakanishi and Whelan 2012). The brain anterior to the cut was removed, and pieces of gel foam were inserted to fill the cavity. At this point, the sciatic nerve and leg muscle groups of the left hindlimb were exposed and their tendons were connected to transducers using methods similar to those described above. Nerve action potentials were recorded using hook‐shaped recording electrodes placed around the tibial and peroneal nerves. Next, anesthesia was stopped while the animal remained on the ventilator (MouseVent, Kent Scientific). Crossed reflex was tested using a moderately strong, brief (<1‐sec) pinch to the right hindpaw (Nakanishi and Whelan 2012). This stimulus caused repeated burst activity in both tibial and peroneal nerves and in both anterior and posterior muscle groups. We termed this reflex action “crossed reflex” because responses were observed in both flexor and extensor muscles of the opposite hindlimb. The tail pinch reflex was induced by a moderately strong, brief (<1‐sec) pinch to the base of the tail. This stimulus produced a strong bilateral reflex that lasted up to 30 sec per bout of activity. The reflex manifested as alternative movements that resembled fast walking, which begins with very quick, strong movements, then slows. tsDCS was applied using the same system described above. To test the short‐term effect of tsDCS, current was applied only during the duration of the reflex, then turned off. To test the long‐term effect of tsDCS, current was applied for 3 min; reflex testing was performed before and every 5 min after tsDCS for 20 min.

### Recording and stimulation in awake animals

An image of the complete system constructed in our laboratory is shown in Figure 1F. The system consisted of a nerve cuff electrode, a tsDCS active electrode, and reference electrodes. These electrodes were connected by wires to a set of four connectors attached by dental cement to the skull. The cuff electrode (Fig. 1G) was fabricated using permanently implantable grade silicone tubing (3 mm long, 1.5‐mm inner diameter, 2‐mm outer diameter; AlliedSil). Wires were made from very flexible stranded stainless steel (10 strands, 280‐*μ*m overall diameter, 45‐Ω/foot impedance, nylon insulation material; Cooner Wire). The wires were guided into the silicone tube using a 27‐gauge needle (Akay 2014). The insulation at the ends of the wires was removed, and a knot was made in each wire to prevent it from slipping into the silicone tubing. The distance between the two wires inside the tubing was about 2 mm. A number 4–0 silk suture (7 cm) was also inserted into the silicone tubing between the two wires. A silicone adhesive was applied over the wire knots, suture, and silicone tubing. Small silicone tubing (5‐mm long, 51‐*μ*m inner diameter) was used to hold the two wires. This tube was also filled with silicone adhesive. The parts were left to dry overnight. Next, the silicone tubing was cut on one side, and the opposite ends of each wire were soldered to a mini connector.

The tsDCS electrodes were fabricated using AlliedSil reinforced permanently implantable silicone sheeting (177‐*μ*m thickness), similar to the electrodes described above for anesthetized animals. The wires used for tsDCS electrodes were the same type as those used for the cuff electrode, and they were also soldered to mini connectors. Once the system was ready for implantation (Fig. 1F), animals were anesthetized using ketamine and xylazine (90/10 mg/kg, i.p.). Animals were shaved at the head, mid‐back, and over the left hindlimb. The surgery was performed in a sterile environment. Three incisions were made over the head, mid‐back, and left hindlimb area above the sciatic nerve. The system was placed in position under the skin. Mini connectors were fixed to the skull with two screws and dental cement. The tsDCS electrode was sutured fabric side down to the fascia of the spine to cover the area from the T13 to the L6 vertebral level. The reference electrode was placed fabric side up toward the lateral abdominal skin. Next, the cuff electrode was brought closer to the sciatic nerve via a cut through the fascia between the gluteus superficialis and biceps femoris muscles. The sciatic nerve was freed from the surrounding fascia and carefully placed into the cuff. The cuff was then sutured to the surrounding muscles. Sterile saline was used to wet the tsDCS electrode before the skin was sutured. Antibiotic was placed over the wound, and animals were placed on a heating pad to recover. Testing started 7 days after surgery. Only animals without walking abnormalities were used for the present experiments. An image of an animal standing on a treadmill with no abnormality is shown in Figure 1H. Sciatic nerve activity recorded during walking is shown in Figure 1I.

### Locomotor testing

The effects of tsDCS on ground and treadmill locomotion were investigated. Before each recording/stimulating session, each animal was placed in a mouse restrainer for about 20 sec to allow connection to the DC stimulator and recording amplifier. During ground walking, animals were allowed to walk freely on an even circular surface area (55‐cm diameter). Animals were also placed on a mouse treadmill at slow (0.2‐m/sec) or fast (1.5‐m/sec) settings to walk. Sciatic nerve activity was recorded before, during, and after tsDCS. In each animal, the effects of short‐term (30‐sec) tsDCS were tested.

### Data analysis and statistics

Units were sorted based on their monophasic spike width, height, and shape using Lab Chart spike histogram software (ADInstruments). The detection threshold was set to target the unit/sec to be analyzed. Spike shape was visually inspected, and spikes were usually viewed at well‐separated clusters using the spike histogram discriminator view. Twitches were assigned to a certain unit spike if they occurred at an appropriate delay (7–8 msec) and based on their repeated occurrence following that specific spike. This delay period was selected based on the time interval between the electrically evoked action potential near or at the site of recoding and its corresponding muscle twitch. Units were then exported to NeuroExplorer software for further analysis. Rasters were constructed for each group of units. A firing rate histogram and auto‐ and cross‐correlograms were created for each group of units. Repeated‐measures analysis of variance (RM ANOVA) was used to compare responses of units over time, with Holm–Sidak post hoc correction to test differences across time points. Mann–Whitney rank sum tests or paired *t*‐tests were used to examine differences between two groups. Pearson correlations were used to analyze correlations between cortically evoked activation and inhibition with firing rate. Pearson correlations were also used to examine the correlation between cortically evoked muscle twitch and gamma motor neuron firing rate. Distribution fitting analysis (Matlab) was used to analyze changes in spike amplitude, frequency, and variance during locomotion. Perievent histograms were used to show the conditional probability of a spike relative to a reference spike. Statistical analyses were performed using SigmaPlot (SPSS, Armonk, NY). Slopes and maximal force were calculated using LabChart software (ADInstruments). NeuroExplorer was used to create auto‐ and cross‐correlograms and perievent and firing‐rate histograms, and to examine coherence. The critical level of significance was set at *P *<* *0.05.

## Results

### tsDCS modulates spontaneous activity of gamma and alpha motor neuron ensemble

**Figure 3:**
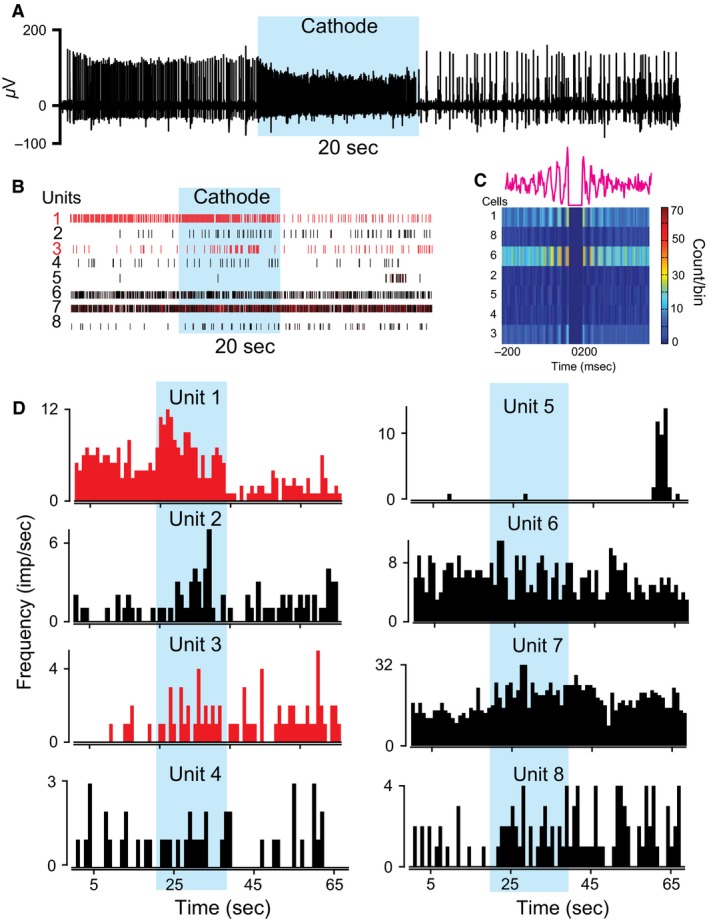
Effects of c‐tsDCS on gamma and alpha motor neuron ensemble. This is the same ensemble as in Figure 2. (A) Raw trace showing unit activity. (B) Rasters of the eight units. (C) The heat map shows cross‐correlograms of all units (unit 7 was the reference). The upper line graph is the cross‐correlogram for unit 1. Note that all units were correlated to unit 7. Note also the rhythmicity in that correlation. (D) Firing rate histograms. Red and black rasters and rate histograms were produced by gamma and alpha motor neurons, respectively. Shaded areas in all graphs indicate recordings during‐tsDCS.

In these experiments, tsDCS was applied for a short duration (20 sec). Figure 2 shows eight single units that were well isolated and classified in a representative animal. Units 1 and 3 were not correlated with muscle twitches and were therefore classified as gamma motor neurons. Based on their autocorrelograms, unit 1 showed a regular firing pattern and unit 3 showed a bursting firing pattern (Fig. 2E) (Bartho et al. 2004). tsDCS caused different responses in these two cells: a‐tsDCS abolished the activity of unit 1, but increased the firing rate of unit 3. Conversely, c‐tsDCS increased the firing rates of units 1 and 3 (Fig. 3). Note that both units showed increased firing rates immediately after the anode was turned off. However, after the cathode was turned off, the firing rate of unit 1 was decreased and the firing rate of unit 3 was increased. Those two units were classified as gamma based on the criteria described in the Methods section. All other units displayed in Figures 2 and 3 were alpha motor units associated with muscle twitches. Motor unit 6 showed a regular firing pattern, and all other motor units showed bursting patterns. During and after a‐tsDCS, firing rates of units 2, 4, and 8 were increased, but firing rates of units 6 and 7 were reduced (Fig. 2D). During and after c‐tsDCS, firing rates of units 2, 4, 7, and 8 were increased, and firing rate of unit 6 was reduced (Fig. 3D). The effect of tsDCS on unit 5 was not determined. Cross‐correlograms showed that all units were co‐activated with unit 7 (Fig. 3C), indicating a common input. However, neurons were modulated differently by tsDCS, which implies that tsDCS affects neurons differently than their input source.

### Overall changes in gamma and alpha motor neuron firing rates during tsDCS

**Figure 4:**
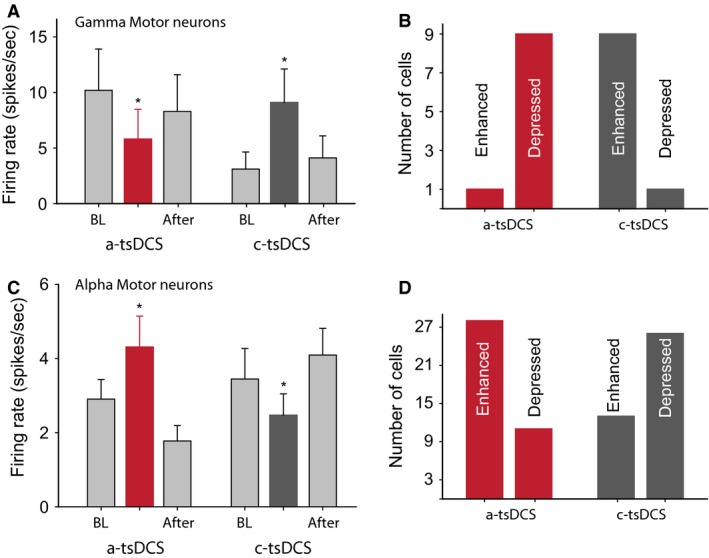
Overall effects of tsDCS on gamma and alpha motor neurons. (A) Summary plot showing that a‐tsDCS decreased gamma motor neuron firing rate, whereas c‐tsDCS had the opposite effect. (B) Gamma motor neurons were divided based on whether they showed enhanced or reduced activity during tsDCS. (C) Summary plot showing the average effect of tsDCS on the firing rate of alpha motor neurons. (D) Alpha motor neurons were divided based on whether they showed enhanced or reduced activity tsDCS. Asterisk indicates significance from baseline (BL). *P < 0.05; data are means ± SE.

To examine overall changes in firing rates, we compared the average firing rates of all neurons before, during, and after tsDCS. Friedman RM ANOVA showed a main inhibitory effect of a‐tsDCS (χ^2^ = 8.4, *P *=* *0.01; Fig. 4A) and a main excitatory effect of c‐tsDCS on gamma motor neurons (χ^2^ = 8.4, *P *=* *0.01; Fig. 4A). During a‐tsDCS of 10 well‐isolated gamma motor neurons (one neuron from each of eight animals and two neurons from one animal), nine neurons showed decreased firing, and one showed increased firing (Fig. 4B). Conversely, during c‐tsDCS of these same gamma motor neurons, nine neurons showed increased firing and one showed decreased firing (Fig. 4B).

RM ANOVA showed a main inhibitory effect of c‐tsDCS (*F *=* *7.2, *P *=* *0.001) and a main excitatory effect of a‐tsDCS on alpha motor neuron firing rate (χ^2^ = 6.7, *P *=* *0.009; Fig. 4C). Neurons were recorded from 15 animals, and at least two well‐isolated neurons from each animal were included in the analysis. During a‐tsDCS of 39 well‐isolated alpha motor neurons, 28 (large units: median force = 0.34 g; median rising slope = 23.9 g/sec) showed increases and 11 (small units: median force = 0.03 g; median rising slope = 2.9 g/sec) showed decreases in mean firing rate (Fig. 4D). Conversely, during c‐tsDCS of these same alpha motor neurons, 13 showed increases and 26 showed decreases in mean firing rate (Fig. 4D). These results indicate that a‐tsDCS and c‐tsDCS have opposite effects on firing rates of both gamma and alpha spinal motor neurons. Furthermore, the twitch force was greater in large alpha motor neurons than in small alpha motor neurons (Mann–Whitney rank sum test, *P *=* *0.001), suggesting that tsDCS affects spinal neurons based on cell size.

### Effects of tsDCS on cortically evoked single motor neuron spikes

#### Cortically activated gamma and alpha motor neurons

**Figure 5:**
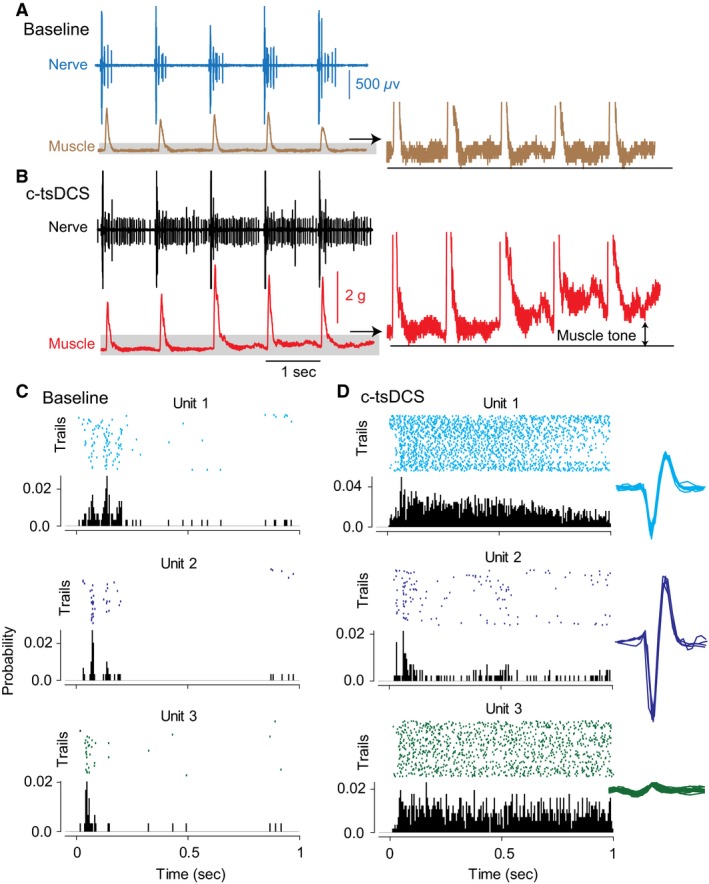
Effects of c‐tsDCS on evoked gamma and alpha motor neuron activity. Cortical stimulation was applied at the M1 area. The stimulus intensity was identical during baseline and c‐tsDCS conditions. (A) Top: five responses from the tibial nerve (blue upper trace) and posterior group muscles (brown lower trace). Shaded area is expanded on the right to show changes in muscle tone. (B) Five responses from nerve and muscle during c‐tsDCS. Note the change in muscle tone during c‐tsDCS. Units 1 and 2 are marked with an asterisk and circle, respectively, in the last trace in A and B. (C) Three units were identified as gamma motor neurons (units 1 and 2) and a tonic alpha motor neuron (unit 3). Perievent raster showing the results of 50 trials: top, trials (each dot corresponds to a spike); bottom, probability histograms. In all graphs using the perievent method, the reference variable is the stimulus artifact of cortical pulses. (D) Perievent rasters of the three units during c‐tsDCS. Right insets showing superimposed raw traces of each of the units.

Using stimulation of M1, we were able to cortically evoke multiple gamma motor units associated with alpha motor units in three animals. Figure 5 shows a robust example of this effect from one animal. Units 1 and 2 were identified as gamma motor neurons (see criteria in Methods), and unit 3 was designated an alpha motor neuron. Cross‐correlograms showed strong interactions among the three units. Autocorrelograms showed that neuron 1 had a bursting pattern with peak activity at 143% of average background activity, which occurred at 33 msec. Neuron 2 had strong bursting activity with peak activity at 1333% of average background, which occurred at 10 msec. Unit 3 had weak bursting activity at 118% of average background, which occurred at 10 msec. Based on the response latency (Ahmed 2011, 2014b), these results suggest different synchronizing input sources to neuron 1 (cortex) than to neurons 2 and 3 (brain stem).

c‐tsDCS increased the firing rates of units 1, 2, and 3 (Fig. 5C and D) compared with baseline. Specifically, unit 1 increased firing from 1.6 ± 0.2 spikes/sec (maximal rate, 4 spikes/sec) to 18.9 ± 1.1 spikes/sec (maximal rate, 30 spikes/sec; paired *t*‐test, *P *=* *0.001). Unit 2 increased firing from 0.9 ± 0.1 spikes/sec (maximal rate, 5 spikes/sec) to 3.7 ± 0.3 spikes/sec (maximal rate 8 spikes/sec; paired *t*‐test, *P *=* *0.001). Unit 3 increased firing from 3.7 ± 0.3 spikes/sec (maximal rate, 11 spikes/sec) to 22.7 ± 1.1 spikes/sec (maximal rate, 40 spikes/sec). c‐tsDCS also increased cortically evoked muscle twitches, and this increase was correlated to the firing rates of unit 1 (*r *=* *33, *P *=* *0.02) and unit 2 (*r *=* *0.32, *P *=* *0.03), but not unit 3 (*r *= −0.03, *P *=* *0.9). These results suggest that independent pathways transmit the supraspinal signals to increase muscle tone and to produce muscle action. The results also suggest that c‐tsDCS increases muscle force production at least partially through the fusimotor system (see Discussion). The effect of a‐tsDCS was not tested in these neurons.

#### Cortically evoked single gamma motor neuron spike

**Figure 6:**
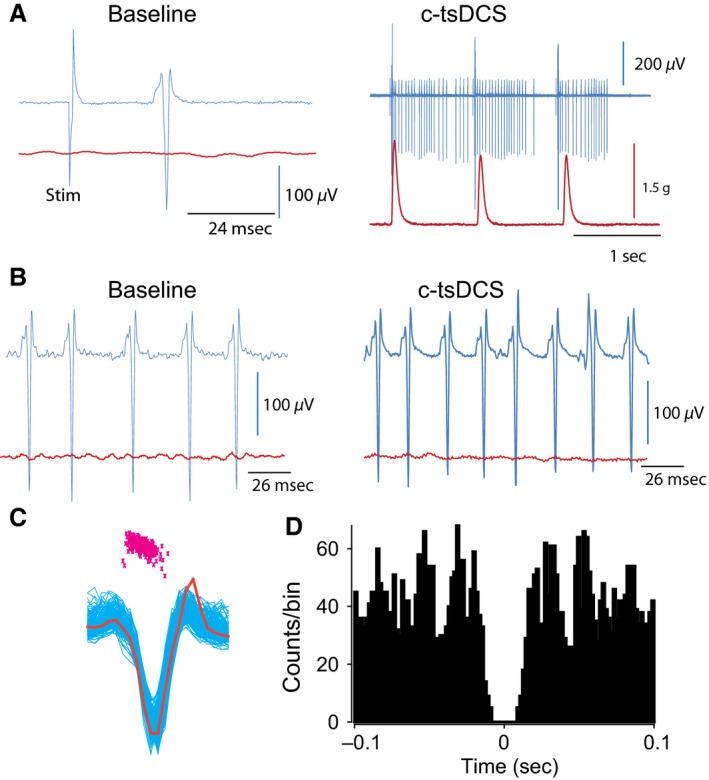
Single gamma motor neuron activity was modulated by c‐tsDCS. (A) Left: at low‐intensity cortical stimulation, a single gamma motor neuron responded. The unit's evoked spike is an appropriate size (270 *μ*V) and shape compared to its spikes during spontaneous activity. No muscle twitch was associated with this unit. Right: when the same unit was cortically evoked during c‐tsDCS, it showed an increase in the firing rate per cortical pulse. (B) The same unit was spontaneously firing. Left: the firing pattern of this unit when c‐tsDCS was off; right: when c‐tsDCS was on. (C) Discriminator view of the neuron spike characteristics. Top: clustering of the unit; bottom: raw traces of the unit with one of the spikes highlighted by the red trace. (D) Autocorrelogram showing that this gamma motor neuron has a rhythmic firing pattern.

**Figure 7:**
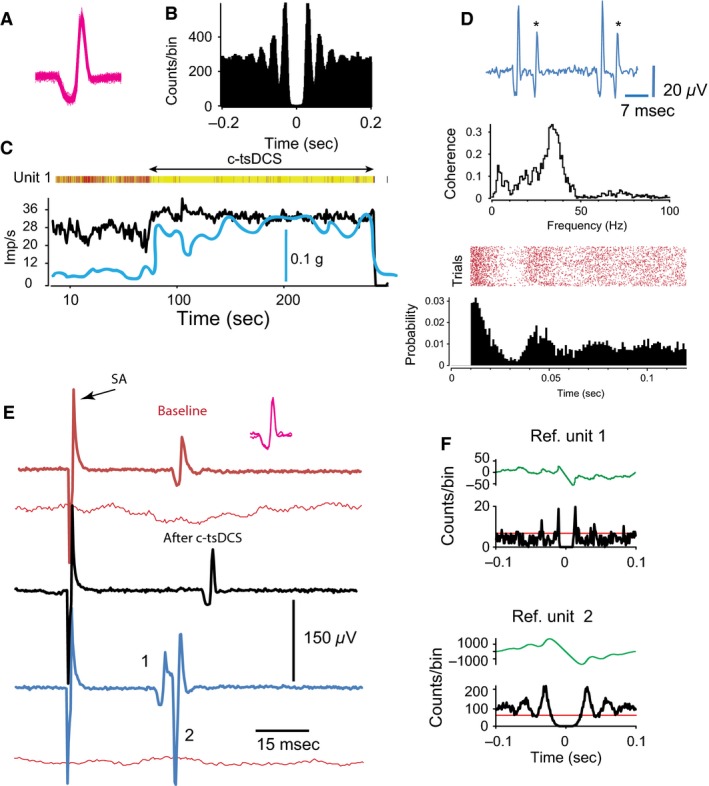
The effects of c‐tsDCS on one gamma motor unit and its associated alpha and gamma motor units. (A) Raw traces for one gamma motor neuron. (B) Autocorrelogram showing the firing features of the neuron shown in (A). Note the rhythmic firing pattern. (C) Top: raster showing the gamma motor neuron spiking pattern before, during, and after c‐tsDCS. Bottom: firing rate of this unit showing a significant increase in firing rate during c‐tsDCS, which did not attenuate. The muscle tone measurement (blue line) is superimposed on the firing rate trace. Note that when firing rate increased, muscle tone was also increased. (D) Activity of a tonic motor neuron associated with the activity of this gamma motor neuron. Top: raw traces of the two neurons; asterisks show spikes of the tonic motor neuron. Middle: coherence analysis showing that activities of those two neurons were coherent at about 44 Hz. Bottom: perievent raster and histogram showing that spike timing of the tonic motor neuron and gamma motor neuron are strongly related. The histogram shows the average delay of the tonic motor neuron is 10 msec. (E) Gamma motor neurons respond to cortical stimulation. Red trace shows response of gamma motor neuron (shown in A) to stimulation of the motor cortex. Note that this neuron was not associated with muscle twitch (lower red trace). Inset shows superimposed raw traces evoked by cortical stimulation. Black trace shows the response of the same neuron to cortical stimulation following 3 min of c‐tsDCS. Note that the neuron responded, but this response was delayed. Bottom: raw trace of two gamma motor neurons (1 and 2) responding to cortical stimulation. Neuron 1 is the same gamma motor neuron shown in A, and neuron 2 is the same gamma neuron described in Figure 6. (F) Cross‐correlograms for a bout of spontaneous activity of the two gamma motor neurons is shown in E (bottom). Cumulative sum graphs are also shown (top, green).

Figure 6 shows the activity of a single gamma motor neuron that was evoked by cortical stimulation (M1; latency, 24 msec). Note that results shown in Figures 6 and 7 were obtained from the same animal (*n* = 1). This neuron fired spontaneously for relatively short periods (about 10 sec), had a bursty autocorrelogram, and could fire at a maximal rate of 44 spikes/sec. Its activity was not correlated with cortically evoked muscle twitch force (*r *=* *0.29, *P *=* *0.4). However, it was indirectly correlated with changes in muscle tone, similar to the gamma motor neuron described in Figure 10. The firing rate of this gamma motor neuron was significantly increased during c‐tsDCS (maximal rate, 34 spikes/sec) compared with baseline (maximal rate, 28 spikes/sec). Cortically evoked spikes were also dramatically increased by c‐tsDCS (maximal rate, 44 spikes/sec) compared with baseline (maximal rate, 1 spike/sec).

In Figure 7, the firing pattern of this single gamma motor neuron is compared with patterns from other gamma and alpha motor neurons. The gamma motor neuron shown in Figure 7A had a bursty, rhythmic autocorrelogram (Fig. 7B). Its activity was indirectly correlated with muscle tone (Fig. 7C). Its firing rate increased during c‐tsDCS (30.1 ± 0.2 spikes/sec) compared with baseline (22.8 ± 0.6 spikes/sec; paired *t*‐test, *P *=* *0.001). During c‐tsDCS, an alpha motor neuron began to fire, increasing muscle tone (Fig. 7D, top). The two neurons abruptly stopped firing after 3.5 min of c‐tsDCS. These alpha and gamma motor neurons had coherence at a frequency of 44 Hz (Fig. 7D, middle). The perievent histogram shows the firing probability of the alpha motor neuron with the gamma motor neuron as the reference point (Fig. 7D, bottom). Figure 7E shows that cortical stimulation (M1) could evoke activity of a single gamma motor neuron (before c‐tsDCS) with a latency of about 23 msec. Although this gamma motor neuron stopped spontaneously firing following c‐tsDCS, it could still be evoked cortically, albeit with a prolonged latency (32 msec; Fig. 7E, middle trace). Unlike the gamma motor neuron described in Figure 6, the spontaneous activity of this gamma motor neuron was not modulated by cortical stimulation. Moreover, c‐tsDCS did not increase cortically evoked discharges of this neuron. These results indicate that the spontaneous activity of this neuron was controlled by a different input source than the cortex, and the two sources were affected differently by c‐tsDCS. Either before or after c‐tsDCS, two gamma motor neurons could be cortically evoked (Fig. 7E, lower trace). Those two gamma motor neurons were cross‐correlated (Fig. 7F). Note that neuron 1 caused peaks at 10 msec (Fig. 7F, top), and neuron 2 caused peaks at 33 msec (Fig. 7F, bottom).

#### Cortically evoked and inhibited alpha motoneuron spikes

**Figure 8:**
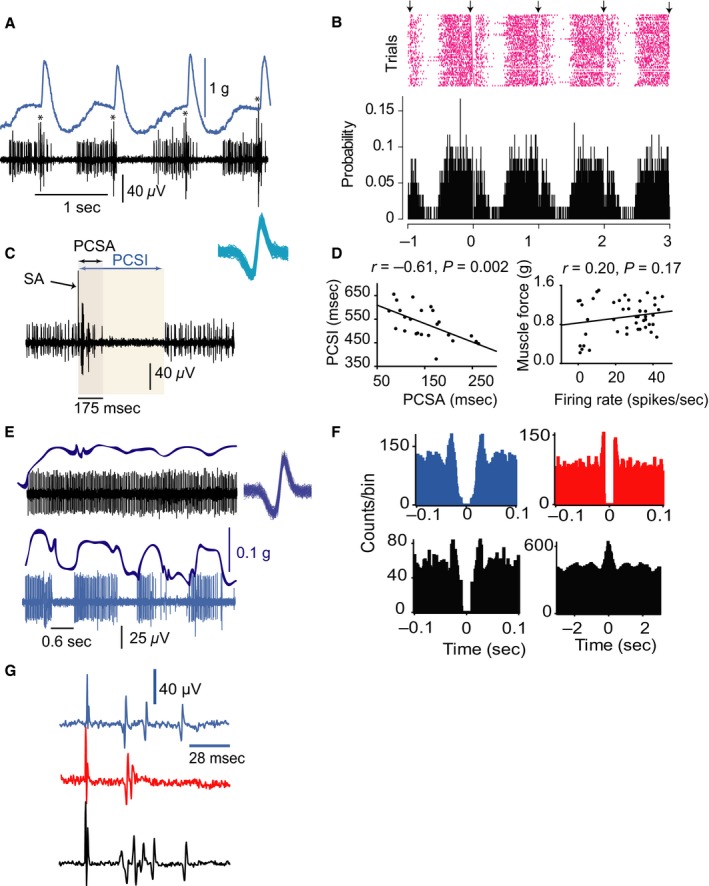
Effects of tsDCS on a tonic alpha motor neuron that was excited and inhibited by cortical stimulation. (A) Tonic discharges were modulated by cortical stimulation. Top: muscle contraction. Bottom: single unit recordings. Asterisks mark the stimulus artifacts. (B) Top: 53 trials showing spikes (dots) following cortical stimulation (arrows). Bottom: perievent histograms showing probability of spikes. Note the suppression of spiking. (C) Measurements of postcortical stimulation activity (PCSA) and inhibition (PCSI) periods. SA, stimulus artifact. Inset showing the superimposed spikes modulated during cortical stimulation. (D) PCSI and PCSA were negatively correlated. The firing rate was not correlated with the amplitude of cortically induced muscle twitches. (E) Top: tonic synchronized spontaneous activity. Muscle contraction is superimposed on nerve activity (blue line). Bottom: slow rhythmic synchronized spontaneous activity of the same neuron. Muscle contraction is superimposed (blue line). Inset on the right side shows superimposed spike traces. (F) Autocorrelograms showing activity pattern during baseline (blue), during a‐tsDCS (red), and during c‐tsDCS (black, left) and extended scale (black, right). (G) Single‐unit activity was evoked by motor cortex stimulation. Traces at baseline (top, blue), during a‐tsDCS (middle, red), and during c‐tsDCS (bottom, black).

Cortically evoked and inhibited alpha motor neurons were observed in two animals (Fig. 8). This type of neuron is especially interesting due to its probable involvement in skilled and unskilled activity, including postural control, locomotion, and adaptive walking. These alpha motor neurons fired in either tonic or slow rhythmic patterns at a maximal rate of 40 Hz. They fired stably for minutes and were subjected to detailed analysis. Autocorrelograms showed a peak at 30 msec, indicating a distant (likely cortical) input source. M1 stimulation could evoke firing of this neuron at a latency of 30 msec and was associated with an immediate postcortical stimulation activity (PCSA) period (152.0 ± 11.1 msec), followed by a postcortical stimulation inhibition (PCSI) period (529.7 ± 14.3 msec), which affected tonic activity (Fig. 8C).

Postcortical stimulation inhibition (PCSI) was negatively correlated with PCSA (*r *= −0.61, *P *=* *0.002; Fig. 8D) and firing rate (*r *= −0.55, *P *=* *0.005). PCSA and firing rate were positively correlated (*r *=* *0.58, *P *=* *0.002). No correlation was observed between the amplitude of cortically evoked muscle twitch and firing rate (*r *=* *0.20, *P *=* *0.17; Fig. 8D), PCSA (*r *=* *0.26, *P *=* *0.22), or PCSI (*r *=* *0.13, *P *=* *0.6). Collectively, these results suggest that cortically evoked spinal responses are mediated by different pathways than those that mediate spontaneous activation or cortically induced inhibition of this type of alpha motor neuron.

Anodal trans‐spinal direct current stimulation (a‐tsDCS) reduced cortically evoked responses (Fig. 8G) and PCSI and increased spontaneous activity, changing it to tonic activity. a‐tsDCS also reduced the latency to peak (10–11 msec) compared with baseline (30–31 msec; Fig. 8F). Since a peak in the autocorrelation can reflect bursting activity, and the postactivation time (from zero) reflects the frequency of bursts (Wichmann et al. 1994), these results indicate a shift in the activation source, or direct influence of anodal stimulation on the intrinsic properties of the neuron.

Cathodal trans‐spinal direct current stimulation (c‐tsDCS) increased cortically evoked responses (Fig. 8G). However, it reduced PCSI and increased spontaneous activity, changing it to rhythmic (1 burst/sec), revealing an influence on the spinal network. c‐tsDCS slightly reduced the latency to peak (26–27 msec; Fig. 8F) compared with baseline (30–31 msec), suggesting presynaptic effects.

### Trans‐spinal direct current modifies crossed reflex and tail‐pinch reflex in decerebrate animals

**Figure 9:**
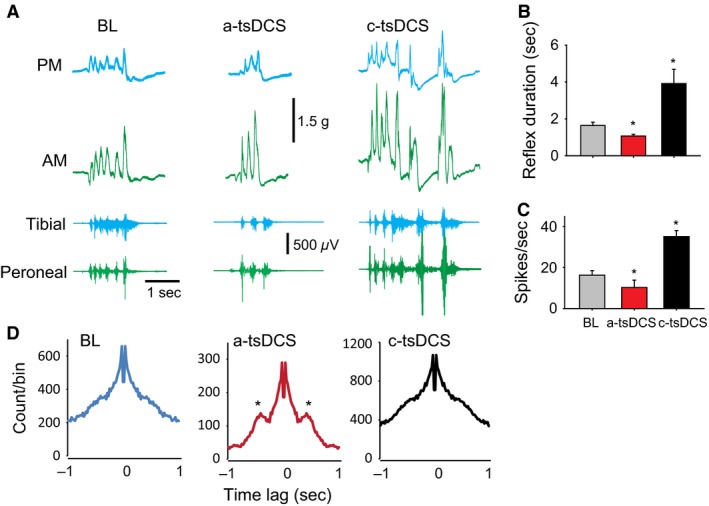
Effect of tsDCS on cross reflex. In decerebrate animals, the right front paw was gently squeezed, producing nerve activity and muscle contraction of the contralateral hindlimb. (A) Examples of muscle contraction (PM, posterior muscles; AM, anterior muscles) and nerve activity at baseline, during a‐tsDCS, and during c‐tsDCS. Note that AM muscles had increased force amplitude during a‐tsDCS. (B) Average duration of reflexes, showing reduction during a‐tsDCS, but enhancement during c‐tsDCS compared with baseline (BL). (C) Average discharge rate, showing reduction during a‐tsDCS, but enhancement during c‐tsDCS compared with baseline. (D) Average autocorrelograms, showing bursting during baseline and c‐tsDCS and bursting and rhythmicity during a‐tsDCS. Note also that background activity decreased during a‐tsDCS and increased during c‐tsDCS. Rhythmicity during a‐tsDCS may explain the increase in AM force. Asterisks mark secondary peaks in the autocorrelogram. *P <* *0.05; data are means ± SE.

Brief (<1‐sec) pinching of the contralateral paw skin causes coordinated movements of the hindlimb in mice (Nakanishi and Whelan 2012). This crossed reflex is a complex movement that is mediated by circuitry within the spinal cord (Rossignol et al. 2006), and it is modulated by descending pathways. The reflex also represents a response to a natural stimulus, in contrast to responses evoked by electrical stimulation of a specific pathway. Thus, the crossed reflex was used in this study to evaluate the effects of tsDCS on local circuit activity. tsDCS significantly modified the crossed reflex (Fig. 9). During a‐tsDCS, both duration and average nerve discharges were reduced compared with baseline values. However, during c‐tsDCS, both duration and average nerve discharges were increased (*n *=* *5; RM ANOVA, *F *=* *14.1, *P *<* *0.001; Holm‐Sidak method, *P *<* *0.01). Muscle twitches mirrored the effects on nerve discharges, except that twitches of the anterior muscle group were increased in amplitude during a‐tsDCS (Fig. 9A). This effect could have contributed directly to changes in synchrony of activity, as reflex responses became more rhythmic during a‐tsDCS, or it could have contributed to the indirect effect of reciprocal facilitation due to inhibition of the antagonist muscles (posterior muscles; Fig. 9A, top). Autocorrelograms showed two important effects of tsDCS: (1) a‐tsDCS reduced overall activity, but made responses more rhythmic, as evidenced by the appearance of two extra peaks in the correlograms (Fig. 9D, middle); and (2) c‐tsDCS doubled the amount of activity, but did not change the rhythmicity of responses (Fig. 9D, right).

**Figure 10:**
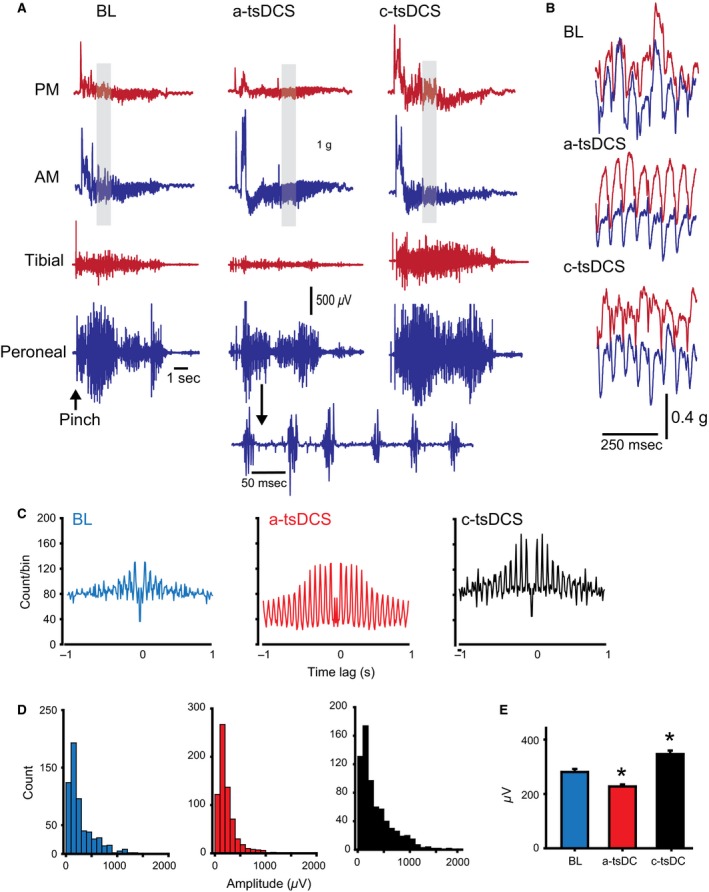
tsDCS modifies tail pinch reflex in decerebrate animals. (A) Examples of tail pinch reflexes recorded during baseline (BL), a‐tsDCS, and c‐tsDCS. Muscle contractions from posterior muscles (PM) and anterior muscles (AM) and nerve discharges from tibial and peroneal nerves were recorded. Pinching the base of the tail in decerebrate animals caused repeated rhythmic movements that continued for about 10 sec. (B) Enlargement of muscle responses (corresponding to highlighted areas in A). Note the rhythmicity of muscle contraction during a‐tsDCS and c‐tsDCS. (C) Average autocorrelograms of peroneal nerve discharges. Note that rhythmicity increased during a‐tsDCS and c‐tsDCS compared with baseline. Note also that activity was generally decreased during a‐tsDCS and increased during c‐tsDCS. (D) Spike amplitude histograms for baseline (left), during a‐tsDCS (middle), and during c‐tsDCS (right). (E) Average spike amplitude. *P <* *0.05.

Next, in a group of decerebrate animals (*n *=* *4), tail pinch (<1 sec) was observed to produce rhythmic movement of both hindlimbs (Fig. 10). Nerve discharges and muscle contractions were recorded as shown in Figure 10. The activity was in the form of bursts with intervals <100 msec (Fig. 10A and B). Reflex activity became more rhythmic during a‐tsDCS and c‐tsDCS compared with baseline (Fig. 10C). Overall activity and spike amplitude were reduced during a‐tsDCS and increased during c‐tsDCS (Fig. 10C and D). Average spike amplitude was also reduced during a‐tsDCS and increased during c‐tsDCS (Fig. 10E; RM ANOVA, *F *=* *35.8, *P *<* *0.001; Holm‐Sidak method, *P *<* *0.001). Interestingly, muscle contraction of the anterior groups was increased despite a reduction in overall nerve activity. These results are similar to those observed in the crossed reflex in a different group of animals (see above). Reduced asynchronized activity (background activity) could improve muscle fiber responses (Fig. 10C) because fewer muscle fibers would be in the refectory period. In addition, disinhibition due to depressed antagonist muscles (posterior muscles) could cause the anterior muscle group to increase muscle force.

Longer (3 min) application of tsDCS had relatively long‐lasting effects on reflex responses. In decerebrate animals (*n *=* *2), 3 min a‐tsDCS or c‐tsDCS elongated the changes observed after short‐term a‐tsDCS or c‐tsDCS (see above), causing them to persist for at least 20 min after stimulation was turned off.

### Effects of tsDCS on locomotion

**Figure 11:**
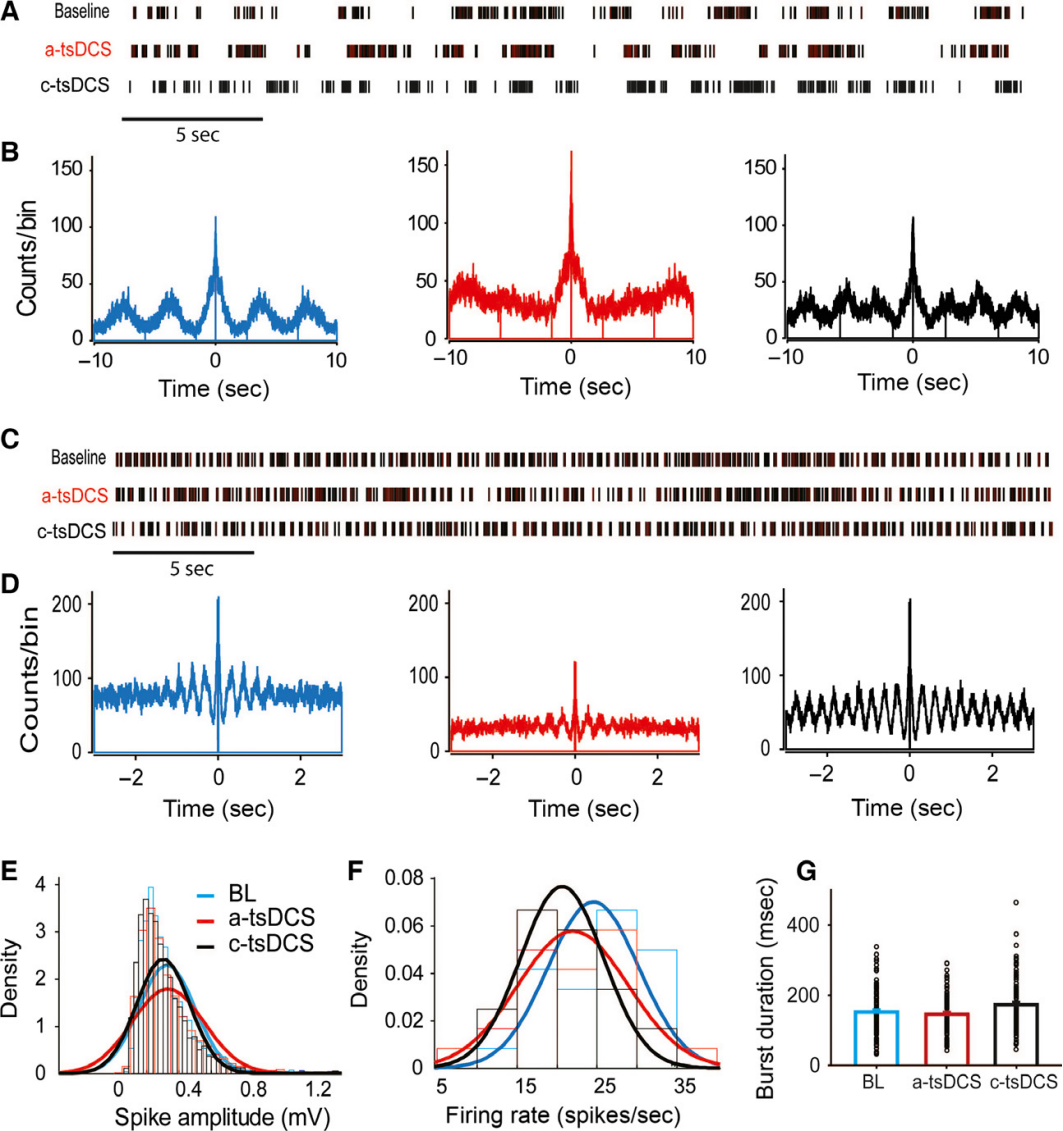
Effects of tsDCS on locomotor activity. (A) Spike rasters of 40 sec of ground walking during baseline, a‐tsDCS, and c‐tsDCS. (B) Autocorrelograms of ground walking during baseline (BL, blue), a‐tsDCS (red), and c‐tsDCS (black). Note the extra peak during c‐tsDCS compared to baseline. (C) Spike rasters of activity during fast treadmill walking. (D) Autocorrelograms of activity recorded during fast treadmill walking under three conditions: baseline (blue), a‐tsDCS (red), and c‐tsDCS (black). (E) Distribution fitting of spike amplitude during baseline, a‐tsDCS, and c‐tsDCS. (F) Distribution fitting of firing rate during baseline (blue), a‐tsDCS (red), and c‐tsDCS (black). (G) Durations of bursts (*n *=* *110) during baseline, a‐tsDCS, and c‐tsDCS. Duration was significantly increased during c‐tsDCS.

To examine the effects of tsDCS on locomotion, animals (*n *=* *9) were implanted with tsDCS electrodes and with cuff recording electrodes around the sciatic nerve (Fig. 1). As shown in Figure 11A and B, a‐tsDCS made bursting during ground walking less rhythmic, and c‐tsDCS increased the number of peaks in the autocorrelograms, indicating more rhythmicity and fast production of bursts. We used a treadmill to standardize walking speed (fast or slow) and to determine how animals would compensate for the extra excitation or inhibition induced by tsDCS. At a fast treadmill speed, a‐tsDCS reduced bursting rhythmicity and c‐tsDCS increased it (Fig. 11D). Similar results were observed at a slow treadmill speed (not shown).

Distribution fitting analysis showed that variance of spike amplitude during treadmill walking was increased during a‐tsDCS (var = 0.05) and reduced during c‐tsDCS (var = 0.02), compared with baseline (var = 0.03; Fig. 11E). This analysis also showed that the firing rate was shifted toward the lower frequency during c‐tsDCS (mean = 20 spikes/sec; var = 27) and the distribution curve was broadened during a‐tsDCS (mean = 22 spikes/sec; var = 47), compared with baseline (mean = 25 spikes/sec; var = 32; Fig. 11F). Next, we examined the effects of tsDCS on burst duration during treadmill running. We compared 110 randomly selected bursts from each treatment condition. RM ANOVA showed that tsDCS had a main effect on burst duration (*F *=* *5.3, *P *=* *0.006; Fig. 11G), which was increased during a‐tsDCS (167.3 ± 6.9 msec, *P *=* *0.01) and slightly but not significantly reduced during c‐tsDCS (140.2 ± 5.4 msec), compared with baseline (146.6 ± 6.1 msec; Holm‐Sidak method, *P *=* *0.4). Collectively, these results indicate that tsDCS can change locomotor rhythm.

## Discussion

This study found several important and functionally relevant effects of tsDCS on spontaneous and cortically evoked gamma and alpha motor neuron single‐unit activity. The main tsDCS effect was cell type specific. That is, responses of gamma motor neurons and smaller alpha motor neurons were generally increased by cathodal, but reduced by anodal, stimulation; larger alpha motor neurons showed the opposite pattern. In addition, tsDCS had functional effects. It modified crossed and tail pinch reflexes, as well as the locomotor activity pattern. These data establish that spinal DCS acts on spinal mechanisms in a predictable manner that can be exploited to enhance motor control and learning.

**Figure 12:**
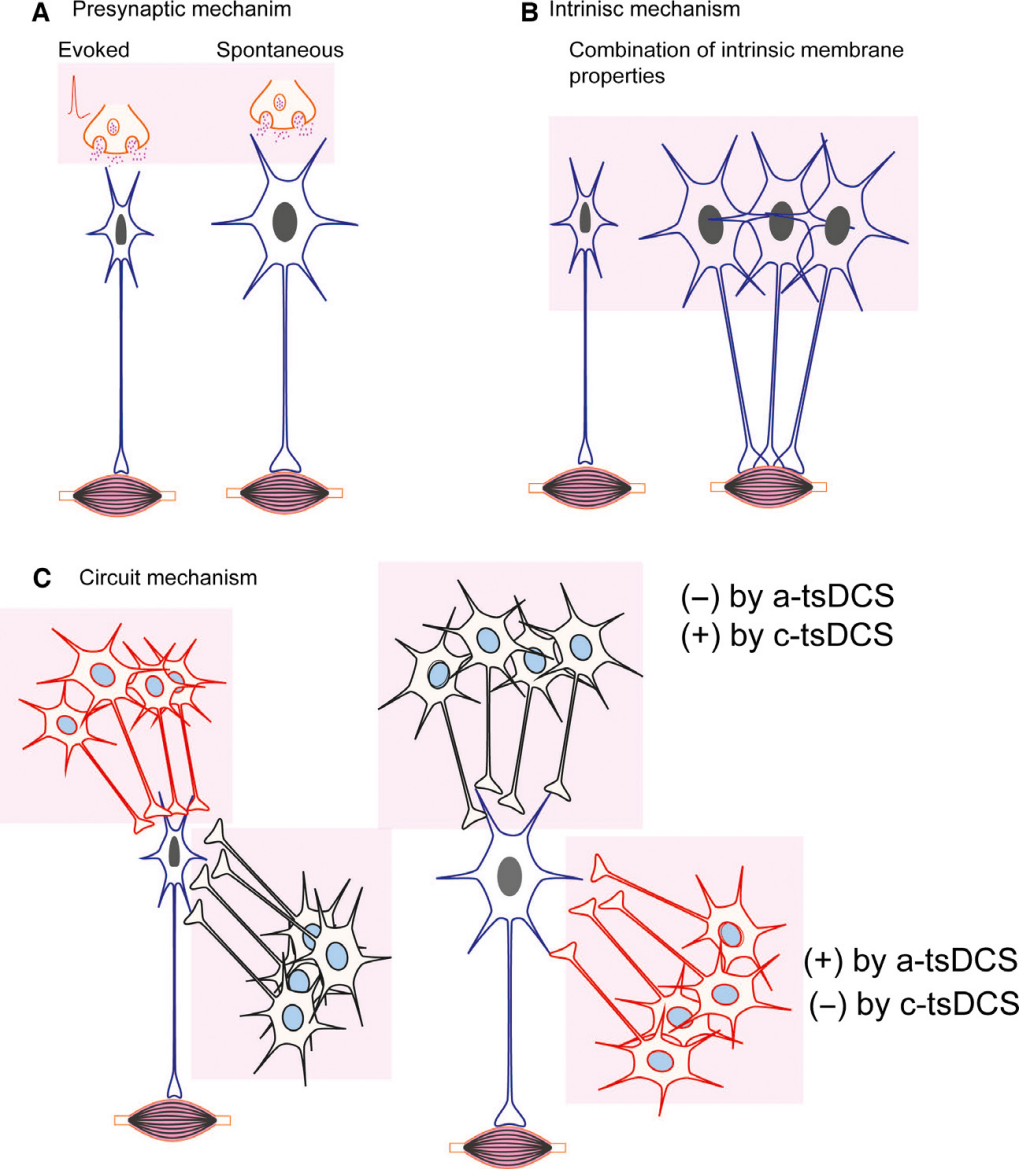
Three mechanisms that could explain the differential effects of tsDCS on spontaneous activity of motor neurons. (A) Presynaptic mechanism, in which tsDCS affects larger motor neurons only through spontaneous presynaptic mechanisms and affects smaller motor neurons only through evoked presynaptic mechanisms. In this case, tsDCS effects could only manifest if the smaller motor neurons were constantly activated by active inputs from presynaptic neurons. (B) Intrinsic mechanism, in which current flow would gravitate toward larger neurons because they have lower input resistance, which would cause them to hyperpolarize or depolarize in response to a‐tsDCS or c‐tsDCS, respectively. The presence of gap junctions between larger neurons would amplify this size effect. Smaller neurons should be less affected due to their higher input resistance. Hyperpolarization‐activated inward currents present in larger motor neurons would cause increased firing rates in response to a‐tsDCS. Other mechanisms would also have to contribute to explain the phenomenon. (C) Circuit mechanism, in which larger and smaller motor neurons are connected, and tsDCS differentially affects populations of spinal interneurons. For example, in response to a‐tsDCS, a population of excitatory premotor neurons (red) oriented as shown in C (left and right) would be excited and inhibited, respectively. This would result from a‐tsDCS induced inward current at the processes and outward current at the cell body (right population) or vice versa (left population). Similar effects could occur at differently oriented inhibitory interneurons (black). Different permutations of these three mechanisms are also possible.

There are three fundamental effects of direct current on neuronal activity and responses. Specifically, c‐tsDCS: (1) increases evoked synaptic transmission; (2) reduces spontaneous synaptic transmission; and (3) depolarizes motor neuron cell bodies. a‐tsDCS has the opposite effects (Eccles et al. 1962; Ahmed 2011, 2013a,b, 2014b; Bolzoni and Jankowska 2015). In this context, “evoked” and “spontaneous” presynaptic activity indicate synaptic release evoked by an action potential or not evoked by an action potential, respectively. Regarding neuronal size, smaller neurons should be more sensitive to tsDCS‐induced changes in synaptic transmission because of their lower threshold and less sensitive to electrotonic spread of current because of their higher input resistance. Conversely, extracellular current should gravitate toward larger motor neurons because of their low input resistance, which should, in turn, make larger motor neurons more likely to be affected by changes to their membrane potentials. However, the data in this study suggest the converse: anodal stimulation increased spontaneous activity in larger motor neurons, and cathodal stimulation reduced spontaneous activity. Thus, the effect of tsDCS on larger motor neurons can be explained by synaptic mechanisms (Fig. 12A). Background activity of larger motor neurons that is mediated by spontaneous synaptic activity should be increased by anodal stimulation and decreased by cathodal stimulation. This implies that the effect of tsDCS on larger motor neurons, seen in this study, can be explained by changes in spontaneous synaptic activity. However, this analogy does not explain the effects of tsDCS on smaller alpha and gamma motor neurons, which are the opposite of the effects on larger motor neurons. The modulatory effect of tsDCS on smaller motor neurons could be explained by changes in synaptic activity evoked by presynaptic action potentials. Thus, if presynaptic spike activity (from spinal or/and brain sources) was not changed in response to tsDCS, but associated postsynaptic responses were modulated, that could explain the effects of tsDCS on smaller motor neurons. In conclusion, the effects of tsDCS on larger motor neurons could be due to tsDCS‐mediated changes on spontaneous presynaptic activity, and its effects on smaller motor neurons could be due to changes in evoked presynaptic inputs. This conclusion is supported by data shown in Figure 8F, in which anodal stimulation reduced latency to the peak of the autocorrelation, but cathodal stimulation did not. Reduction in the latency to peak indicates changes in the postburst period (Gullo et al. 2009), which reflect direct changes in the source of the activation. This activation must be either local or due to spontaneous synaptic activity. Conversely, cathodal stimulation increased activity, but did not change the pattern or the latency to peak, indicating that the source was not local and must therefore have been due to evoked synaptic activity. This analysis does not explain why larger motor neurons would preferentially respond to spontaneous but not evoked synaptic activity and vice versa for smaller motor neurons. However, this could be explained if different mechanisms predominate in different types of neurons. For example, if evoked presynaptic activity is predominant in smaller motor neurons due to stronger tonic inputs from spinal or brain sources, this would lead to manifestation of evoked presynaptic mechanisms in these neurons even if the spontaneous presynaptic mechanisms were still operational.

Another possible mechanism could be that membranes of larger motor neurons have a unique expression profile of hyperpolarization‐activated cation channels (Anderson et al. 2012) that would increase their spontaneous activity when exposed to hyperpolarizing anodal DC current, analogous to neurons in other regions (Akasu et al. 1993; Zhang et al. 2003). This would be more effective in larger motor neurons because of the lower input resistance, which would make them the path of least resistance for extracellular applied current. The effect could be amplified in larger motor neurons containing gap junctions, which would lower their input resistance (see review by Kiehn and Tresch (2002) and increase their spontaneous activity during a‐tsDCS even further (Fig. 12B). This hypothesis is supported by unpublished data from our lab showing that calcium uptake was increased during tsDCS in synaptosomes. A third possible explanation could be differential effects at spinal interneurons (Fig. 12C). Spinal excitatory and inhibitory interneurons have unique connectivity patterns with motor neurons (McLean et al. 2007, 2008) (see also a review by Goulding (2009)). In addition, the presence of gap junctions between premotor interneurons (see review by Kiehn and Tresch (2002)) could amplify the effects of tsDCS. tsDCS could differentially modulate spinal interneurons based on polarity, causing size‐based tsDCS effects on motor neurons (Fig. 12C) if distinct populations of spinal interneurons are aligned differently relative to the applied field (see Fig. 12). If neurons were aligned randomly to the direction of current, there would be zero net effect. A situation is depicted in Figure 12C in which groups of premotor neurons are arranged in opposite directions around larger and smaller motor neurons. In this situation, a‐tsDCS would have opposite effects on inhibitory interneurons (black) oriented in opposite directions, inhibiting their input to larger motor neurons, but exciting their input to smaller motor neurons. Similarly, excitatory interneurons oriented in opposite directions around smaller and larger motor neurons could cause opposite effects. Another likely mechanism is the involvement of the Renshaw cells. The inhomogeneous synaptic connections between RCs and motor neurons (Windhorst and Koehler 1983) would partly explain the effect size of tsDCS in alpha motor neurons.

The results presented in Figures 5, 6, and 7, as well as the strong cross‐correlation shown in Figure 2C, demonstrate that the primary motor cortex co‐activates alpha and gamma motor neurons. This result supports previous studies (Sjostrom and Zangger 1975; Dimitriou and Edin 2010). That is, gamma motor neurons can adjust movement production in a feedforward manner, in addition to the classical feedback role through muscle spindles (Vallbo 1981). More importantly, the present data show that the gain of this feedforward mechanism of motor control can be modified using tsDCS. Another important observation from these experiments was the lack of correlation between muscle tone and cortically evoked muscle force. This finding supports our previous conclusion that cortically evoked alpha motor neuron output is not due to feedback input. Moreover, different mechanisms are involved in producing movement and adjusting muscle tone during movement. This finding also provides a mechanism for the hypothesis that spasticity is not correlated with muscle strength (Rymer and Katz 1994). More importantly, these results provide a mechanism for c‐tsDCS–induced increases in cortically evoked actions (Ahmed 2013a, 2014a).

The gamma motor neurons described in Figures 6 and 7 provide strong evidence of differential effects of c‐tsDCS on presynaptic input to gamma motor neurons. c‐tsDCS increased both spontaneous and cortically evoked firing of the gamma motor neuron described in Figure 6. However, c‐tsDCS increased only spontaneous, not cortically evoked, activity of the gamma motor neuron described in Figure 7. These results indicate clearly that presynaptic inputs to certain gamma motor neurons are specifically modulated by c‐tsDCS.

Both c‐tsDCS and a‐tsDCS reduced PCSI (Fig. 8), indicating direct effects on spinal inhibitory interneurons. However, c‐tsDCS and a‐tsDCS could induce this effect through different mechanisms. Given that a‐tsDCS reduced cortical activation of this unit, reduction in PCSI could be due to a presynaptic depression of cortical inputs. Moreover, a direct or indirect influence via increase in excitability of this unit by a‐tsDCS could also be a factor. Given that c‐tsDCS increased cortically evoked discharges from the unit, reduction of PCSI could be due to an increase in excitatory cortical or subcortical inputs. Activation and inhibition of this unit by the same cortical stimulus could be a correlate for postural tone adjustment associated with voluntary movements (Massion 1984). Therefore, tsDCS effects on this unit, investigated in this study, could be of great interest to explore clinically. For example, increasing the firing rate and reducing PCSI could be beneficial in conditions requiring precise action (Kakuda et al. 1996). However, it should be emphasized that the above conclusions were made based on recordings obtained from fewer number of neurons.

Tail and paw pinching in decerebrate animals has been shown to produce rhythmic movements in both hind limbs (Fig. 9 and 10). Despite a rather brief duration of pinching, the subsequent motor activity outlasted the stimulus by 30 sec in some animals, especially following tail pinch. This indicated that tail pinching served as a triggering event to shift the spinal network into perpetuating cycles of autogenic stimulation. Tail or paw pinching has been shown to facilitate locomotor activity in animals with spinal cord injury (Sherrington 1910; Meisel and Rakerd 1982; Pearson and Rossignol 1991; Majczynski et al. 2005; Slawinska et al. 2012). Modulation of these complex reflexes by tsDCS, as shown in the current experiments, is a significant finding. It provides direct evidence that tsDCS: (1) modulates spinal centers for locomotion; and (2) can modulate responses to sensory inputs (touch or proprioception), which can be a significant factor in promoting recovery after spinal cord injury (Pearson 2004; Rossignol et al. 2008). tsDCS showed two main effects on tail pinch reflex: (1) c‐tsDCS increased and a‐tsDCS decreased burst amplitude; and (2) both a‐tsDCS and c‐tsDCS caused the pattern to become more rhythmic. This suggests that tsDCS differentially affects mechanisms controlling burst amplitude and rhythmicity of activity and that a‐tsDCS would have an inhibitory effect on spinal inhibitory interneurons. This would improve rhythmicity by reducing the influence of inhibitory spinal interneurons, akin to the effect of strychnine. It could also reduce burst amplitude by reducing inputs from excitatory spinal interneurons (premotor). However, this does not exclude other direct mechanisms of reducing burst amplitude, such as reducing synaptic transmission between afferents and motor neurons (Eccles et al. 1962). In contrast, c‐tsDCS increased burst amplitude and improved rhythmicity (Fig. 10 C). This supports our prior results that c‐tsDCS modulated strychnine‐induced fast rhythmic activity (Ahmed 2013a), indicating that c‐tsDCS has a direct effect on spinal locomotor networks.

Treadmill walking is a rhythmic pattern of activity only when the speed of the walker matches the speed of the treadmill. Slowing below the speed of the treadmill would cause the walker to drift backward. To compensate, the walker would have to walk faster than the treadmill speed. If repeated, this would cause a nonrhythmic locomotor pattern. This analogy can explain the results from the locomotion experiments (Fig. 11). c‐tsDCS made the locomotor activity pattern more rhythmic, meaning that it made the walking speed more similar to the treadmill speed. a‐tsDCS, however, caused a mismatch between the two speeds, thereby reducing rhythmicity of the locomotor activity pattern. These findings raise the question of how tsDCS causes these changes. There are three possible explanations: (1) c‐tsDCS increases while a‐tsDCS decreases the sensitivity of the fusimotor system, as shown in this study, which would change the feedback (Vallbo 1981) and feedforward (Dimitriou and Edin 2010) functions of the fusimotor system. That is, c‐tsDCS would enable the animal to match the speed of the treadmill by correctly predicting the sensory consequences of stepping; (2) tsDCS alters the output of the rhythm generator (Rybak et al. 2006). This hypothesis is supported by the reflex experiments shown in this study (Figs. 9 and 10) and by our previous study (Ahmed 2013a). Directly modifying the temporal pattern of the rhythm generator circuits in the spinal cord would cause an increase or decrease in locomotion speed; (3) tsDCS modifies the sensorimotor integration at the level of the spinal cord. Timing and amplitude of stepping could be modified by proprioceptive inputs from the limbs (Hiebert et al. 1996). c‐tsDCS increases and a‐tsDCS decreases afferent mediated H‐reflex (Ahmed and Wieraszko 2012; Ahmed 2014a); thus, tsDCS could modulate proprioceptive input. Factors 1, 2, and 3 may also contribute to the observed locomotor changes.

a‐tsDCS decreases and c‐tsDCS increases supraspinal (Ahmed 2011) and externally evoked spinal activity (Aguilar et al. 2011; Ahmed and Wieraszko 2012). Taken together, one of the major conclusions of this study is that online effects of c‐tsDCS reduce the spontaneous‐to‐evoked activity ratio, and a‐tsDCS increases this ratio. This finding has implications for the clinical application of tsDCS. Given that reductions of spontaneous‐to‐evoked activity ratio can switch the learning rules from internally to externally driven (Tritsch et al. 2007; Toyoizumi et al. 2013), c‐tsDCS could theoretically enhance motor learning of a task performed during c‐tsDCS. Task performance during a‐tsDCS should cause the opposite effect. These conclusions suggest that tsDCS would allow artificial control of the amount of learning of a sequential task (e.g., learning how to drive). Therefore, externally applied direct current could manipulate the neuronal state of activity similar to how endogenous inhibitory mechanisms (e.g., GABA circuits) function to change the sensitivity of neuronal networks to experiences (Tritsch et al. 2007).

Clearly, the polarity of tsDCS influenced sensorimotor transformation (Figs. 9 and 10). General findings to date indicate that c‐tsDCS magnifies extrinsic (e.g., touch) and intrinsic (Ia afferent) sensory inputs, whereas a‐tsDCS reduces them. Therefore, control of tasks that depend heavily on this sensorimotor transformation (e.g., walking, bicycling, and reflexive movements) would be expected to change. The results of our locomotor experiments suggest that increasing the sensory input gain improves the speed and quality of walking. This hypothesis supports the idea that sensory activity level increases as a function of task difficulty (Prochazka et al. 1988). In conclusion, the findings in this study present a deeper understanding of the physiological effects of spinal DCS. This knowledge could be exploited to carefully design intervention protocols to modulate motor control and learning.

## Conflict of Interest

None declared.
